# Supplementary material for: Salmonella-Based Therapy Targeting Indoleamine 2,3-Dioxygenase Restructures the Immune Contexture to Improve Checkpoint Blockade Efficacy
Source: Biomedicines. 2020 Dec 16;8(12):617. doi: 10.3390/biomedicines8120617 (PMC7765568; doi:10.3390/biomedicines8120617)
Supplement: Supplementary file 1 [file biomedicines-08-00617-s001.pdf]

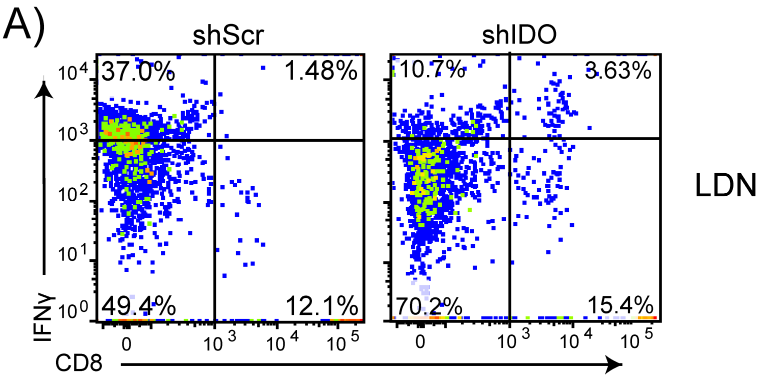

Figure S1. Phenotypes of SIINFEKL-specific CD8+ splenocytes after incubation with neutrophils induced by shScr or shIDO-ST. (A) Flow cytometry dot plots of activated CD8 T cells (CD8 and IFNγ double positive) out of total OTI splenocytes after 24 hours of in vitro co-incubation with fixed, SIINFEKL-loaded LDN from either shScr or shIDO-ST treated mice.

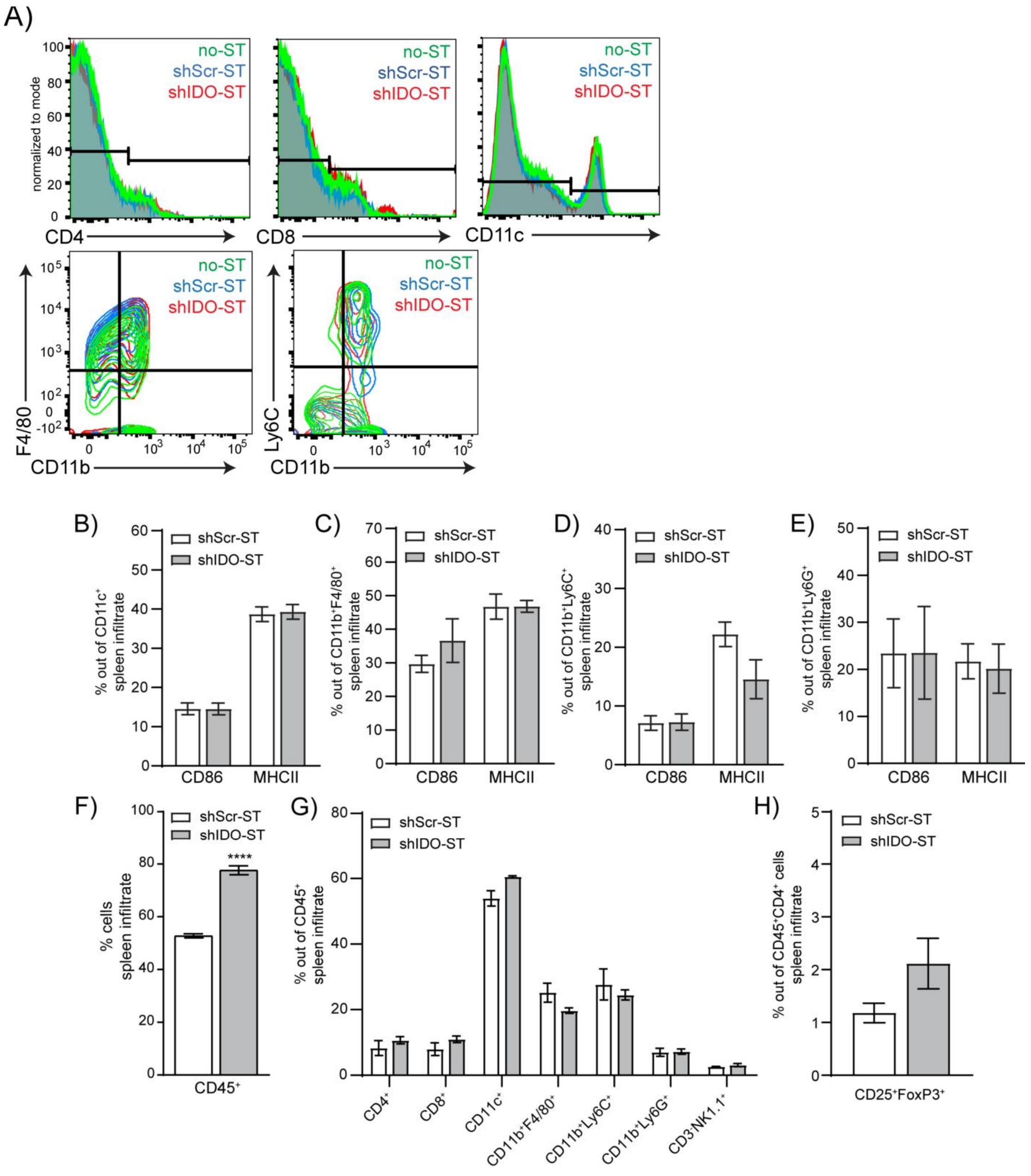

Figure S2. Phenotypes and frequencies of splenocytes in tumor-bearing mice after shIDO-ST treatment. Six days after implantation of LLC1 cells into mice, mice were treated with three consecutive, daily doses of 1 × 10^6 cfu shScr or shIDO-ST. Spleens and tumors were processed 48 hours after the third treatment. All bar graphs represent quantifications of flow cytometry. (A) Flow histograms of total cell types from $CD45^+$ tumor infiltrate. Percentages of CD86 or MHCII positive cells by immune cell type were quantified (B) out of $CD11c^+$ cells, (C) out of $CD11b^+F4/80^+$ cells, (D) out of $CD11b^+Ly6C^+$ cells, and (E) out of $CD11b^+Ly6G^+$ cells. (F) Total $CD45^+$ cells and (G) individual cell types out of total $CD45^+$ splenocytes were quantified. (H) The percentage of splenic Tregs ($CD25^+Foxp3+$) was quantified out of $CD45^+CD4^+$ cells. *$p < 0.05$, **$p < 0.01$, ***$p < 0.001$, ****$p < 0.0001$.

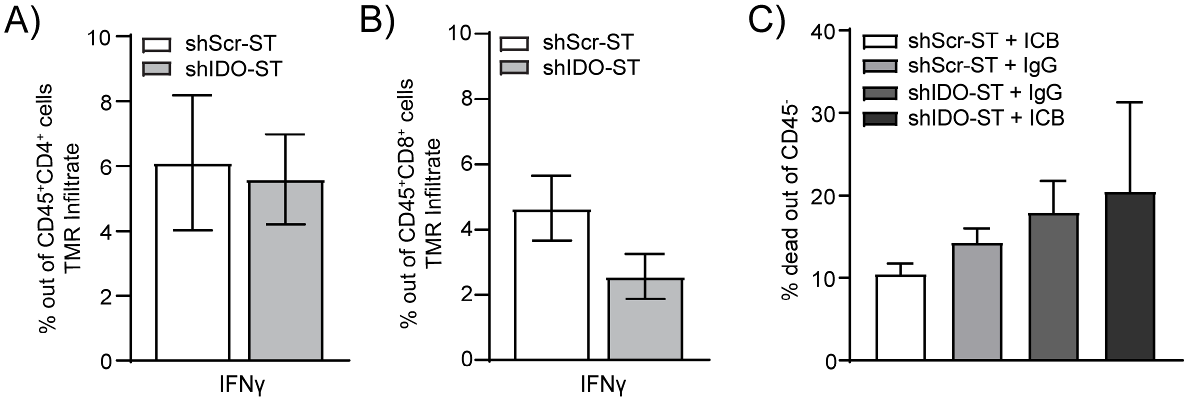

Figure S3. Activation of intratumoral T cells after shIDO-ST treatment. Six days after implantation of LLC1 cells, mice were treated with three consecutive, daily doses of 1 × 10^6 cfu shScr-ST or shIDO-ST. Tumors were processed 48 hours after the third treatment. All bar graphs represent quantifications of flow cytometry. Percentages of IFNγ positive cells by immune cell type were quantified (A) out of $CD4^+$ cells and (B) out of $CD8^+$ cells. (C) Mice with palpable LLC1 tumors were treated with three consecutive doses of 1 × 10^6 cfu of shScr or shIDO-ST combined with immune checkpoint blockade (ICB includes both anti-PD-1 and anti-CTLA-4) or IgG isotype control. Anti-PD-1 was administered at a dose of 200 μg and anti-CTLA-4 was administered at a dose of 75μg every three days until endpoint (max. tumor diameter of ~15 mm). N = 4–10 mice per group. Bar graphs represent flow cytometric analysis of dead cells out of CD45 negative cells 48 hours after the third ST treatment (24 hours after the second ICB or IgG treatment).
